# Supplementary material for: Stacking Model‐Based Classifiers for Dealing With Multiple Sets of Noisy Labels
Source: Biom J. 2025 Mar 12;67(2):e70042. doi: 10.1002/bimj.70042 (PMC11898607; doi:10.1002/bimj.70042)
Supplement: Supplementary file 1 — Supporting Information [file BIMJ-67-e70042-s001.zip › density-based-ensemble-model-main/competitors_real_data/netlab-master/netlab-master/help/sompak.htm]

Netlab Reference Manual sompak


# sompak

## Purpose

Combines node weights into one weights matrix.

## Synopsis

```
c = sompak(net)
```

## Description

`c = sompak(net)` takes a SOM data structure `net` and
combines the node weights into a matrix of centres
`c` where each row represents the node vector.

The ordering of the parameters in `w` is defined by the indexing of the
multi-dimensional array `net.map`.

## See Also

`som`, `somunpak`

---

**Pages:**
Index


---

Copyright (c) Ian T Nabney (1996-9)
